# A Three-Dimensional Biomimetic In Vitro Model to Simulate Schwann Cell-Mediated Peripheral Nerve Repair

**DOI:** 10.3390/gels12070605

**Published:** 2026-07-07

**Authors:** Kristina Pinkham, Amelia Ridolfo, Avantika Jain, Koyal Garg

**Affiliations:** 1 Department of Biomedical Engineering, Saint Louis University School of Science and Engineering, Saint Louis, MO 63103, USA; kristina.pinkham@slu.edu (K.P.); mia.ridolfo@slu.edu (A.R.); 2 Department of Pharmacology and Physiology, Saint Louis University School of Medicine, Saint Louis, MO 63103, USA; avantika.jain@slu.edu

**Keywords:** Schwann cells, spheroids, neurogenesis, bioscaffold, in vitro modeling, hydrogel, lyophilization

## Abstract

Peripheral nerve injuries represent significant clinical challenges, often resulting in lifelong motor function loss and disability. Evidence suggests regenerating axons cannot cross nerve gaps without Schwann Cell (SC) assistance. However, FDA-approved biomaterial conduits for peripheral nerve repair lack bioactivity and structural complexity needed to facilitate SC migration. To address this, we developed a three-dimensional biomimetic in vitro model to simulate complex cellular interactions within the nerve bridge. The model features lyophilized hydrogel bioscaffolds with longitudinal channels to recapitulate the nerve microenvironment. To encourage directional SC dispersion, the top ~30% of the bioscaffold was conjugated with macrophage inflammatory protein-1α (MIP-1α). Rat SCs were seeded within no-MIP-1α- and MIP-1α-conjugated bioscaffold channels as spheroids and cultured for nine days. Histology demonstrated MIP-1α conjugation retained more SC spheroids during culture with greater cellular distributions. SC spheroid culture in MIP-1α-conjugated bioscaffold resulted in enhanced paracrine signaling characterized by increases in VEGF, ICAM-1, IL-6, and CINC-1 production, alongside downregulation of IL-1β, IL-10, and IL-13. The SC-derived pro-inflammatory and pro-angiogenic mediators did not inhibit NSC-34 motor neurite extension compared to controls. This study establishes an in vitro model that serves as both a screening platform and mechanistic tool, advancing our understanding of peripheral nerve repair.

## 1. Introduction

The peripheral nervous system (PNS) is composed of a mixture of motor and sensory nerves, which contribute to motor function and movement output [1]. Peripheral nerve injury (PNI) is a common occurrence following accidents in civil and military populations, which affects 2–5% of trauma patients worldwide [2,3,4,5,6]. After PNI, the regenerative window remains open only for a limited time. If robust regeneration is not achieved within this critical period, prolonged denervation can lead to demyelination of the nerves, resulting in detrimental and often permanent motor deficits [7,8,9,10]. This is especially crucial in complete transection injuries, known as laceration PNI or LPNI, as these injuries are clinically significant, result in extremely challenging recovery, and can lead to diminished quality of life [2,5].

Schwann cells (SCs) are responsible for myelinating large-diameter axons, are vital for saltatory conduction, and are the main mediators of peripheral nerve regeneration [4,7,11,12]. Directly following LPNI, damaged axons in the nerve bridge begin to break down in a process known as Wallerian degeneration, in which SCs respond to the degenerating axon signals and become reprogrammed into a progenitor-like phenotype that has multiple roles throughout nerve regeneration [8,13,14,15]. Reprogrammed SCs first assist ganglia macrophages in the controlled breakdown of myelin and axonal debris; then, SCs dedifferentiate a second time, where they migrate as cellular cords along regenerating nerve vasculature and support axon growth across the nerve bridge [13,15]. SC cord migration is guided by directional chemoattractant signaling from the chemokine CCL3, also known as macrophage inflammatory protein-1α (MIP-1α), which is secreted by hypoxic macrophages in the nerve bridge following laceration nerve injury and forms a chemotactic gradient [15]. Previous studies have shown that MIP-1α chemotactic signaling following nerve injury is required for functional nerve regeneration, as the loss of MIP-1α causes SCs to become misdirected, leading to less efficient migration and guidance of regenerating axons [15].

It has been demonstrated that migrating cords of SCs cannot navigate through the nerve bridge matrix if nerve vasculature has been compromised, indicating how SC-mediated regeneration may not be feasible without surgical intervention in cases of LPNI [15,16]. Traditional nerve grafting surgical treatments, such as autografts, face many limitations in feasibility and efficacy, often leading to substantial donor-site deficits and complications [16]. To combat these limitations, biomaterial nerve conduits (BNCs) are a promising approach, as they alleviate donor-site morbidity concerns and are widely available [5,10,16,17,18]. Current BNCs are made from a variety of materials, ranging from synthetic polymers to decellularized natural proteins, and often contain guiding pores, microchannels, and specific patterning to further encourage axon regeneration by providing physical support [7,9,10,16,17,18,19]. These scaffolds are primarily first- and second-generation biomaterials, as they provide physical support for the regenerating nerve and may be resorbable [16]. BNCs have shown great success in clinical applications, as evidenced by their FDA approval, and have demonstrated improvements for nerve grafting surgery, such as improving surgical time and reducing difficulty [16]. However, BNCs have primarily enhanced functional efficacy in small nerve defects, with further research needed to progress treatments in more complex, larger nerve deficits [16].

Despite the success of current BNCs for nerve regeneration, FDA-approved nerve conduits lack directional biosignaling cues [13,16]. To more closely mimic native nerve tissue, models incorporating extracellular matrix (ECM) proteins, trophic factors, and cellular components can improve treatment efficacy for small and large nerve deficits alike, leading to the development of enhanced third-generation BNCs [4,16,20]. Our lab has previously established a biomimetic, lyophilized hydrogel scaffold composed of type 1 collagen, laminin-111, and type A gelatin crosslinked with 1-ethyl-3-(3-dimethylaminopropyl) carbodiimide (EDC) and N-hydroxysuccinimide (NHS), which has demonstrated regenerative capabilities in skeletal muscle injuries [21]. Modifying the established biomimetic scaffold with guidance microchannels will mimic the native nerve fascicle structure and provide physical support for cells within the matrix while satisfying biocompatibility considerations. Furthermore, the addition of MIP-1α to the bioscaffold will provide the chemical signaling required to direct and initiate SC migration, which we aim to replicate. Implementing cellular components into this bioscaffold, such as culturing SCs, will further enhance regenerative capabilities and support biosignaling pathways [16]. Seeding the SCs as cellular spheroids will more closely mimic native cellular density and increase their regenerative potential [8]. This has been demonstrated by a previous study utilizing injection of SCs in spheroidal geometry into a murine sciatic nerve injury model, which found enhanced pro-repair SC phenotype acquisition and regenerative potential [8].

To improve current treatment options, a three-dimensional in vitro model of SC-mediated peripheral nerve regeneration is needed to better understand SC-mediated nerve repair. Using an in vitro system to model the nerve bridge during regeneration, we can identify the required chemical and physical guidance cues that current BNCs and traditional nerve models may be lacking, leading to less efficient functional outcomes. The main goal of this study is to create a three-dimensional biomimetic in vitro model of peripheral nerve regeneration utilizing a lyophilized hydrogel conduit composed of ECM proteins and longitudinal channels for physical guidance, a spatial MIP-1α functionalization for chemical guidance, and seeded SC spheroids for cellular investigation. We hypothesize that the creation of longitudinal channels through the bioscaffold, coupled with a localized MIP-1α chemotactic zone, will provide both physical and chemical guidance cues to support the directional growth and dispersion of SCs through the bioscaffold’s matrix to closely replicate native nerve repair.

## 2. Results and Discussion

To assess the feasibility of the experimental model and examine the SC paracrine potential, a variety of in vitro analyses were conducted. Formation of the BioGroove scaffold channels was verified using scanning electron microscopy (SEM) imaging. Model feasibility and optimization were conducted using primary rat SCs. Subsequent experiments were conducted with a rat SC line (RT4-D6P2T; ATCC). Spheroids were assessed for viability after four days of culture using a Live/Dead assay. Quantification of DAPI^+^ Area Fraction (%) and spheroid presence was determined via histological analysis. The cellular distribution in seeded channels was examined by stitching together fluorescent histological images and performing Fast Fourier Transform (FFT) analysis. Characterization of the SC secretome in response to MIP-1α conjugation was performed using cytokine arrays and appropriate enzyme-linked immunosorbent assays (ELISAs) on collected culture supernatants. Analysis of neurite differentiation and extension in response to Schwann cell-conditioned media was also performed.

### 2.1. Results

#### 2.1.1. BioGroove Scaffold Characterization

**Figure 1:**
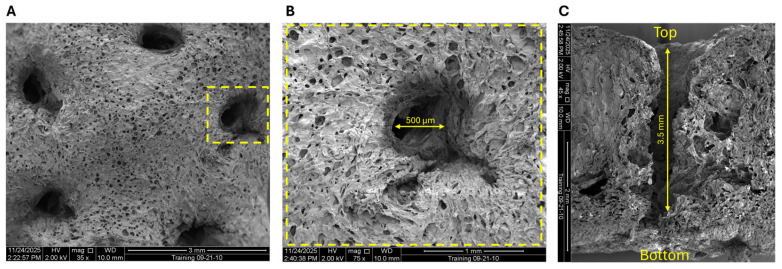
SEM of lyophilized BioGroove Scaffolds at (**A**) 35× magnification, (**B**) 75× magnification, and (**C**) a transverse cross section at 45× magnification to demonstrate channel diameter and depth.

SEM visualization of BioGroove scaffolds revealed that channels remain throughout the scaffold sterilization and lyophilization process, verifying successful GelGrooving (Figure 1). Channels were visually consistent in size and shape across the surface of each observed bioscaffold, demonstrating that sufficient repetitions of GelGrooving had been achieved using the device (Figure 1A). Quantification of BioGroove channels yielded an approximate diameter of 500 µm, which corresponds to the schematic design of the GelGroover device and confirms that the channels did not collapse throughout the drying process (Figure 1B). Transverse cross-sections of BioGroove scaffolds revealed that channels had obtained a depth of 3.5–4.5 mm (~70% of 5–7 mm bioscaffold height) (Figure 1C). The transverse view also showed that the GelGroover device did not remove ECM material during channel formation but instead pushed it aside.

#### 2.1.2. Spheroid Viability Assay

**Figure 2:**
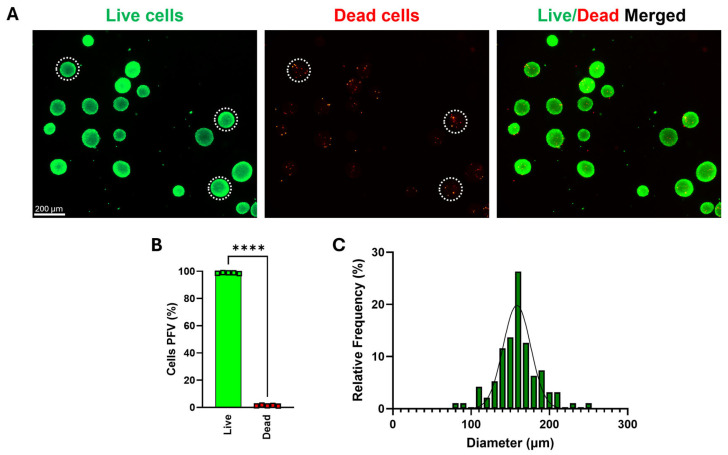
Live/Dead assay analysis of cell-line SC spheroids at day 4 of culture to assess spheroid viability. (**A**) 5× magnification images of stained spheroids with the Live/Dead assay kit, with the green regions representing the alive cells and the red regions representing the dead cells; scale bar = 200 µm, *n* = 5 wells. (**B**) Spheroid viability (%) per field of view (PFV) in the 5× images. (**C**) Histogram of spheroid diameter measured from the Live/Dead images. **** indicates significance (*p* < 0.0001).

Spheroid viability at four days of culture was assessed utilizing a Live/Dead assay (Figure 2A). The percentage of living cells was significantly higher (*p* < 0.0001) than that of dead cells, indicating viability throughout spheroid formation (Figure 2B). In addition, the average viability and the average percentage of dead cells were calculated from the per-field-of-view (PFV) percentages and were approximately 98.75% and 1.25%, respectively. The diameter of four-day cultured spheroids was also determined, and the average spheroid diameter was determined to be 159.35 µm (Figure 2C).

#### 2.1.3. Establishing Model Feasibility Utilizing Primary Schwann Cells

**Figure 3:**
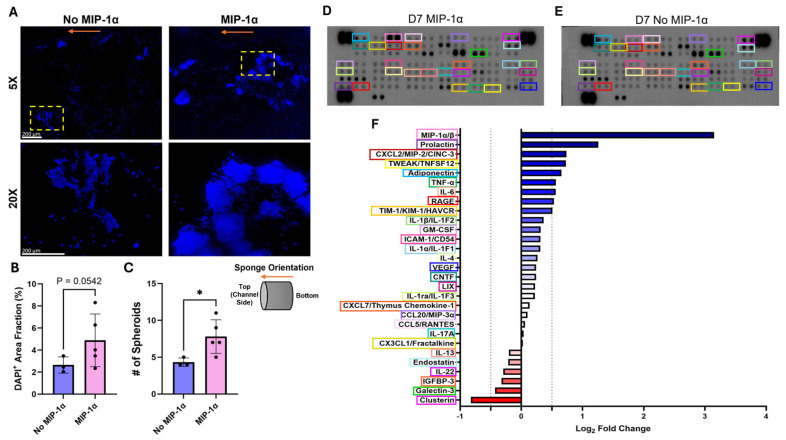
(**A**–**C**) DAPI staining and quantification of BioGroove scaffolds seeded with primary rat SC spheroids (D9), *n* = 3–5 scaffolds/group. (**A**) 5× and 20× images, yellow outline represents the 20× region of interest, orange arrow represents the upward direction of the seeded channels; scale bars—200 µm. (**B**) DAPI Area Fraction (%) per field of view of 10× DAPI images. (**C**) Numbers of spheroids quantified per field of view of the 5× images. “*” indicates significance. (**D**,**E**) Cytokine array of pooled day 7 (D7) cell culture supernatants from MIP-1α- and non-MIP-1α-conjugated bioscaffolds. (**F**) Quantification of band density expressed as log_2_ fold change over the non-MIP-1α-conjugated bioscaffolds. Corresponding color boxes around the specific secretion factors in (**F**) are located on the membranes (**D**,**E**) to demonstrate antibody location.

Following successful spheroid formation, preserved primary SC spheroid-seeded scaffolds were imaged at various magnifications for quantification following nine days of culture (Figure 3). Representative histological images of the DAPI-stained channels show few to no spheroids but numerous single cells in the non-MIP-1α-conjugated scaffolds. In contrast, well-retained and organized spheroids were observed in the MIP-1α-conjugated scaffolds (Figure 3A). When quantified, MIP-1α-conjugated scaffolds tend to show a higher incidence of identified DAPI^+^ nuclei in the BioGroove channels than the non-conjugated controls, although this is not significant (*p* = 0.0542) (Figure 3B). Additionally, the number of spheroids quantified was significantly greater in response to the MIP-1α conjugation than in the non-conjugated controls (*p* = 0.0459) (Figure 3C).

To assess trophic factor secretion in response to MIP-1α conjugation, an exploratory proteome profiling of D7 primary cell supernatants was performed (Figure 3). Membranes were treated with detection antibody solution containing either a pooled MIP-1α supernatant (Figure 3D) or a pooled non-MIP-1α supernatant (Figure 3E) from the primary SC culture. As these preliminary findings represent a pooled sample, statistical analysis could not be performed. However, descriptive log_2_ fold change quantification revealed that MIP-1α-conjugated bioscaffolds activated the SCs, leading to an increase (>0.5 fold change) in Prolactin, CXCL2, TWEAK, Adiponectin, TNF-α, and IL-6 production, alongside a downregulation (<−0.5 fold change) of Clusterin, Galectin-3, IGFBP-3, IL-22, Endostatin, and IL-13 (Figure 3F), relative to the unconjugated scaffold.

#### 2.1.4. Cellular Quantification and Analysis

**Figure 4:**
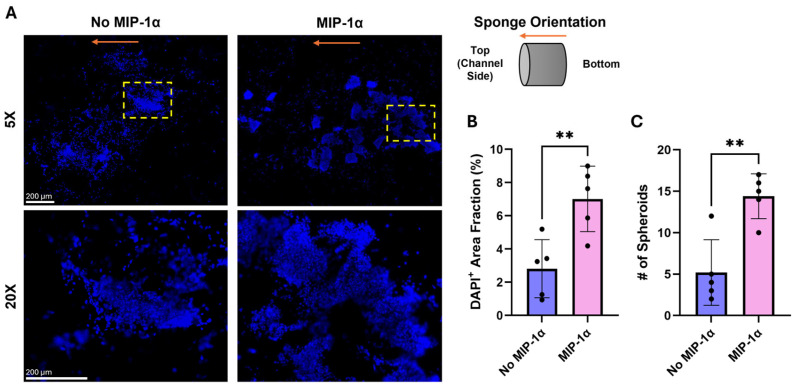
DAPI staining and quantification of BioGroove scaffolds seeded with SC spheroids on day 9 of culture. (**A**) 5× and 20× images, yellow outline represents the 20× region of interest, orange arrow represents the upward direction of the seeded channels; scale bars—200 µm, *n* = 5 scaffolds/group. (**B**) DAPI Area Fraction (%) per field of view of 10× DAPI images. (**C**) Number of spheroids quantified per field of view of the 5× images. ** indicates significance (*p* < 0.01).

All subsequent experiments were conducted using the rat SC line (RT4-D6P2T; ATCC). Following nine days of culture, preserved SC cell-line spheroid seeded bioscaffolds were imaged at various magnifications for quantification (Figure 4). Representative histological images depict unorganized cellular content in the non-MIP-1α-conjugated bioscaffolds and well-retained spheroids in the MIP-1α-conjugated bioscaffolds following day 9 of culture (Figure 4A). MIP-1α-conjugated bioscaffolds contained significantly (*p* = 0.0075) greater DAPI^+^ nuclei in the BioGroove channels compared to the non-conjugated controls (Figure 4B), indicative of both increased cell survival and/or proliferation. Similar to DAPI^+^ area fraction, the number of spheroids was significantly (*p* = 0.0035) greater in response to the MIP-1α conjugation than in the non-conjugated controls (Figure 4C). These results verify increased spheroid retention in the presence of MIP-1α and further indicate enhanced paracrine capabilities in the SC population due to the chemokine conjugation.

#### 2.1.5. Cellular Distribution Analysis

**Figure 5:**
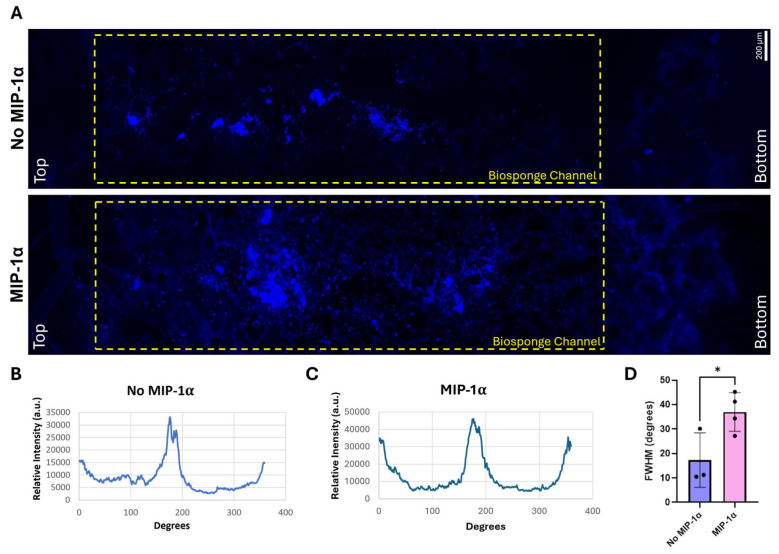
(**A**) Stitched 5× images showing cellular distribution in MIP-1α- and non-MIP-1α-conjugated channels; scale bar = 200 µm. (**B**) FFT analysis of non-MIP-1α-conjugated channel distribution. (**C**) FFT analysis of MIP-1α-conjugated channel distribution. (**D**) Full width at half maximum (FWHM) analysis of FFT data, *n* = 3–4 scaffolds/group. The asterisk symbol (*) denotes statistical significance (*p* < 0.05).

To visualize cellular distribution throughout the BioGroove channels, a series of 5× magnification DAPI images was taken of the SC cell line-seeded spheroids on day 9 (Figure 5). Stitched channel images revealed individual cells exhibiting vertical and lateral dissemination from the spheroids in both experimental groups (Figure 5A). Additionally, Fast Fourier Transform (FFT) analysis revealed a single discrete peak in the no-MIP-1α group, indicating that the cells remained sequestered within the primary longitudinal channel (Figure 5B). However, in the MIP-1α group, three distinct FFT peaks suggest that the cells showed significant lateral infiltration into the surrounding bioscaffold matrix (Figure 5C). The multi-peak profile indicates a divergence from the initial seeding axis, reflecting enhanced cellular dispersion. This observation is quantitatively supported by the angular distribution profiles, where the unconjugated controls exhibited a tightly restricted alignment with an average full width at half maximum (FWHM) of 17°, whereas the MIP-1α-conjugated scaffolds demonstrated a significantly broader cellular distribution, more than doubling the average spread to 37° (*t*-test, *p* = 0.0399) (Figure 5D) [22].

#### 2.1.6. SC Spheroid Secretome Characterization

**Figure 6:**
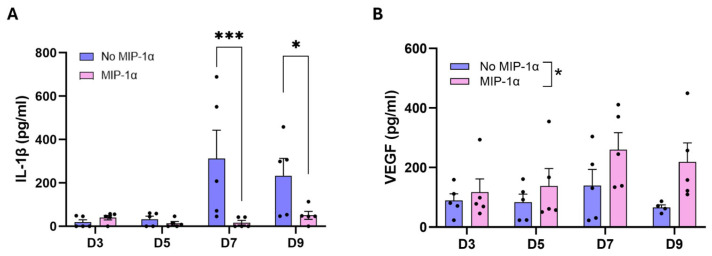
Quantification of secreted factors from SC line-seeded BioGroove Scaffolds using ELISA. (**A**) IL-1β ELISA quantification and (**B**) VEGF ELISA quantification. * indicates significance (* *p* < 0.05, *** *p* < 0.001), *n* = 5 scaffolds/group.

Secretion of vascular endothelial growth factor (VEGF) and interleukin-1β (IL-1β) was determined on days 3, 5, 7, and 9 to further characterize cellular response in the BioGroove scaffolds (Figure 6). The release of IL-1β from SCs in cell culture supernatants was quantified (interaction *p* = 0.0245). IL-1β production was significantly increased on day 7 (post hoc, *p* = 0.006) and day 9 (post hoc, *p* = 0.0257) in the non-MIP-1α-conjugated bioscaffolds, indicating a pro-inflammatory response (Figure 6). Furthermore, ELISA quantification of VEGF showed a significant increase between treatment groups (column factor, *p* = 0.0125) but no significant interaction (*p* = 0.5415), further suggesting an inflammatory response in the SCs due to MIP-1α exposure (Figure 6B). The significance of IL-1β- and VEGF-quantified results strongly indicate MIP-1α conjugation enhanced reparative secretions in the seeded SCs spheroids, peaking around day 7 of culture.

**Figure 7:**
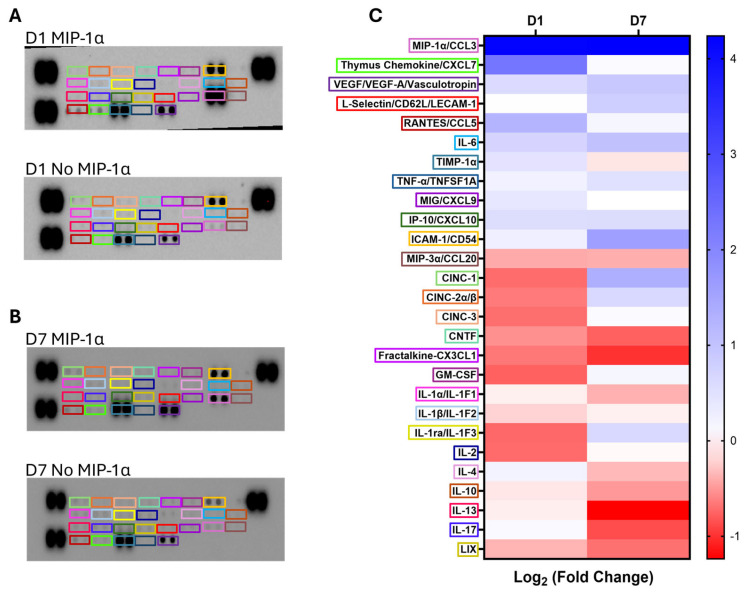
Cytokine array of pooled day 1 (D1) and day 7 (D7) supernatants from seeded BioGroove scaffold culture to compare early inflammatory time points. (**A**) D1 MIP-1α-conjugated membrane and non-MIP-1α-conjugated membrane. (**B**) D7 MIP-1α-conjugated membrane and non-MIP-1α-conjugated membrane. Corresponding color boxes around the specific secretion factors in (**C**) are located on the membranes (**A**,**B**) to demonstrate antibody location.

To further assess trophic factor secretion in response to MIP-1α conjugation, exploratory proteome profiling of day 1 (Figure 7A) and day 7 (Figure 7B) pooled cell-culture supernatants was performed. While statistical analysis could not be performed, descriptive log_2_ fold changes are provided. The release of MIP-1α from the conjugated bioscaffolds showed a roughly 4 log_2_ fold increase over the unconjugated ones on both days 1 and 7, indicating a persistently elevated presence of the chemokine in the cellular microenvironment. Quantification also revealed that MIP-1α-conjugated bioscaffolds activated the SCs, leading to an increase (>0.5 fold change) in Thymus Chemokine, RANTES (Regulated on Activation Normal T-Cell Expressed and Secreted), IL-6, and VEGF, alongside a downregulation (<−0.5 fold change) of GM-CSF (Granulocyte–Macrophage Colony-Stimulating Factor), IL-1ra (Interleukin-1 Receptor Antagonist), and IL-2 at day 1 (Figure 7C). Quantification also revealed that MIP-1α-conjugated bioscaffolds continued to activate the SCs, leading to an increase (>0.5 log_2_ fold change) in ICAM-1 (Intercellular Adhesion Molecule-1), CINC-1 (Cytokine-induced Neutrophil Chemoattract 1), IL-6, and VEGF, alongside a downregulation (<−0.5 log_2_ fold change) of IL-13, Fractalkine, and IL-17 at day 7 (Figure 7C). These secreted factors are indicative of an early pro-repair response from the SCs in the presence of MIP-1α.

#### 2.1.7. Motor Neuron Extension

**Figure 8:**
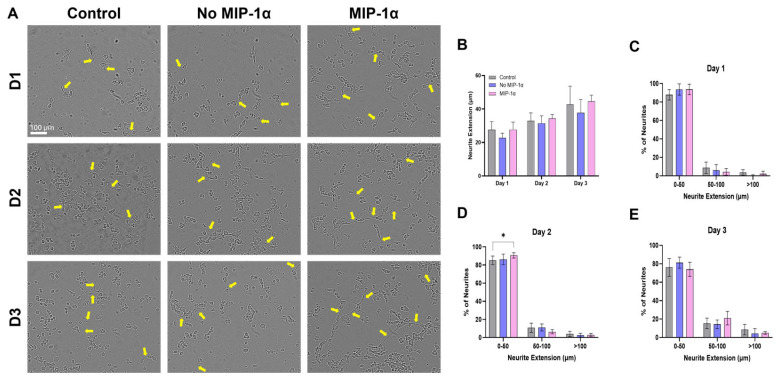
(**A**) NSC-34 cells were differentiated in three groups (Control: regular differentiation media; No MIP-1α: 50/50 differentiation media with non-conjugated supernatant; MIP-1α: 50/50 differentiation media with conjugated supernatant) to examine elongation in response to D9 SC secretome; scale bar = 100 µm, *n* = 5 wells/group. (**B**) Elongation throughout the entirety of the culture duration and a (**C**–**E**) size-bin extension breakdown of each individual day. * indicates significance (*p* < 0.05).

Neuroregenerative potential in response to SC secretome was examined using a motor neurite extension assay by utilizing the Mouse Motor Neuron-Like Hybrid Cell Line (NSC-34; RRID: CVCL_D356; CLU140, Cedarlane Cellutions Biosystems Inc.) (Figure 8). NSC-34 cells were differentiated in three different groups of media: positive control with regular differentiation media, a 50/50 mix of non-MIP-1α-conjugated media with regular differentiation media, and a 50/50 mix of MIP-1α-conjugated media with regular differentiation media (Figure 8A). While the mean neurite extension increased significantly over time (*p* < 0.0001), no significant differences were observed between treatment groups (interaction *p* = 0.9351), suggesting that the treatment had no effect on the rate of outgrowth (Figure 8B). Treatment did not alter neurite size distributions on days 1 and 3 but did on day 2 (interaction *p* = 0.0398) in the smallest size bin (Figure 8C–E). At this time point, MIP-1α-conjugated treatment significantly increased the frequency of neurites in the smallest size bin (0–50 µm) relative to controls. No significant differences between the differentiation groups suggest that MIP-1α exposure does not hinder neurite growth despite its inflammatory nature.

### 2.2. Discussion

To address the spatial and biosignaling requirements of peripheral nerve regeneration, this study developed a 3D in vitro platform. A primary challenge in neuroengineering is replicating the anisotropic architecture of native nerve fascicles [23,24,25]. Without directional cues, regenerating axons disperse, leading to poor functional recovery and neuroma formation. It is well established in peripheral nerve tissue engineering that aligned topographical architectures perform significantly better than random, isotropic matrices [26,27,28]. By employing a straightforward technique, we successfully created longitudinal microchannels within a 3D lyophilized ECM hydrogel. This simple approach achieves biomimicry without complex equipment or scalability limitations often associated with microfluidic systems [29] or high-resolution 3D printing [30]. In characterizing the lyophilized hydrogel, SEM imaging demonstrated that the GelGroover device successfully created longitudinal channels for cell seeding with sufficient diameter and depth for spheroids.

Another key feature of the in vitro platform was the consideration of cellular density and microenvironmental signaling. Traditional models utilizing 2D monolayers or cell-seeded 3D hydrogels fail to capture the cell-to-cell signaling at the injury site. To overcome this limitation, we introduced SCs in the form of high-density spheroids. This approach effectively mimics the dense packing of cells observed within the Bands of Büngner during Wallerian degeneration [31]. By grouping the cells into spheroids, we expected to positively modulate cell survival, proliferation, and the collective migratory behaviors essential for bridging large nerve gaps. SCs were aggregated into spheroids (600 cells/spheroid), seeded into the BioGroove scaffold channels, and cultured for nine days in vitro. SC spheroids have been shown to increase ECM protein deposition and cell–ECM interactions when compared to single-cell suspensions [8]. A previous study assessed SC spheroid viability within the 120 µm range, but the spheroids used in this model were larger in diameter and cultured for longer, so a Live/Dead assay was necessary [8]. Proof of viability is crucial, as large (>200 µm) 3D spheroidal geometries often restrict the diffusion of nutrients, oxygen, and growth factors into the spheroid core during long-term culture, which may induce cell death within the innermost regions, known as necrotic core formation [32].

To provide localized biochemical signaling, we conjugated MIP-1α directly to the lyophilized hydrogel. Following peripheral nerve injury, the sequential recruitment and activation of cells are primarily driven by chemokine gradients. Although the release kinetics was not determined, we hypothesize that immobilizing MIP-1α through chemical crosslinking likely prevented the rapid, burst-release diffusion typical of soluble factors, thereby maintaining a sustained availability of MIP-1α within the bioscaffold. Histological analysis of seeded SC spheroids demonstrated significantly more identified nuclei in MIP-1α-conjugated channels as well as higher retention of spheroid morphology. The cellular distribution between the unconjugated and conjugated scaffolds differed. Quantitatively, the unconjugated scaffolds restricted Schwann cell alignment to a narrow average FWHM of 17°, while the MIP-1α-conjugated scaffolds significantly broadened this distribution to an average FWHM of 37°. The spatial transition from highly localized confinement in the control group to widespread distribution across a broader geographical region suggests higher cellular infiltration into the conjugated scaffolds. Although cellular migration was not directly assessed through conventional techniques due to technical limitations, these data provide indirect evidence of enhanced cell motility. These results suggest that MIP-1α immobilization provides stabilizing cues that reinforce cell–matrix and cell–cell adhesion, resulting in a more conducive environment for SC growth.

Currently, biochemical functionalization strategies for nerve guidance conduits (NGCs) have overwhelmingly relied on the immobilization of traditional neurotrophic growth factors (such as NGF or GDNF) or anti-inflammatory interleukins (such as IL-4) aimed at suppressing localized immunoreactivity [33,34]. While these approaches support localized axonal sprouting, they fail to actively engage the resident supporting cells or guide their spatial routing across the critical defect gap. Furthermore, while the passive delivery of standard monocyte chemokines (e.g., CCL2) has been explored to induce generalized macrophage recruitment [35], the covalent tethering of C-C motif chemokine ligand 3 (CCL3/MIP-1a) as an integrated architectural driver remains virtually unexplored in neural biomaterials design. While the present study demonstrates a clear divergence in Schwann cell behavior upon exposure to MIP-1α-conjugated bioscaffolds compared to unmodified controls, a distinct soluble MIP-1α delivery group was not evaluated. In vitro, soluble chemokines typically undergo rapid passive diffusion, forming a homogeneous concentration profile, while also undergoing accelerated proteolytic degradation. In contrast, the covalent immobilization strategy employed here was designed to structurally anchor the chemokine to the bioscaffold to provide stable, long-term haptotactic signaling.

Beyond mimicking physical architecture and supporting cellular proliferation and dispersion, this engineered microenvironment also modulated the secretory profile of the SCs. Peripheral nerve repair requires a coordinated cascade of biochemical signals to orchestrate cellular recruitment, growth, differentiation, and maturation [36]. In our model, cells cultured within the MIP-1α-conjugated bioscaffolds released significantly higher levels of trophic factors specifically associated with inflammation and angiogenesis from both sources. This enhanced secretome is highly relevant to the in vivo injury response, as localized inflammatory signals are required to promote macrophage infiltration, while angiogenic factors stimulate the neovascularization necessary to support axonal migration and cellular survival [37].

SC line culture supernatants collected and pooled on day 1 revealed the highest expressions of Thymus Chemokine, RANTES, IL-6, and VEGF, which are indicative of early inflammation due to MIP-1α conjugation. Thymus chemokines are heavily involved in neural-immune crosstalk mechanisms, including the modulation of T-cell recruitment and thymocyte migration to nerve injury sites [38]. RANTES, or CCL5, is secreted by SCs following nerve transection injuries and contributes to various nerve repair processes, including macrophage recruitment, immune cell recruitment, Wallerian degeneration, SC regulation, and promotion of nerve growth [35]. IL-6 is secreted by activated SCs and plays a key role in the regulation of immune responses, acute-phase responses, and inflammatory processes following nerve injury, further demonstrating that SCs are activated by this system [39,40]. Additionally, there was upregulation of VEGF on day 1, indicating a pro-reparative, angiogenic response throughout the entirety of culture, aligning with the ELISA findings. While the secretion of these factors suggests a pro-inflammatory and pro-angiogenic response, further in vitro and in vivo assays are required to confirm their exact roles on specific cell types and tissue repair.

The cytokine array also suggested successful MIP-1α conjugation, as it showed the greatest increase in fold change on day 1, which persisted through day 7. However, we did not determine the passive release kinetics of MIP-1α from acellular scaffolds in this study, nor did we perform a dose–response evaluation of varying chemokine concentrations. Consequently, we acknowledge the possibility that exogenous immobilization of MIP-1α may have increased endogenous MIP-1α/CCL3 secretion through a potential positive autocrine or paracrine feedback loop mediated by the engineered matrix. Therefore, the elevated levels of MIP-1α may be attributed to release from both bioscaffolds and SCs.

Quantification of pooled day 7 cell-line supernatant revealed the greatest upregulation of ICAM-1, CINC-1, IL-6, and VEGF, indicative of early inflammation and pro-angiogenic signaling due to MIP-1α conjugation. ICAM-1 is a cell-surface adhesion receptor known for its role in leukocyte trafficking and driving inflammation in epithelial injury-resolution responses [41,42]. In PNIs, ICAM-1 recruits endothelial cells to the nerve gap following injury. CINC-1 is typically upregulated following ischemia and helps to mediate neutrophil infiltration following nerve injury, also contributing to inflammation [43]. IL-6 was found to be persistently elevated on both days 1 and 7. IL-6 is also known to upregulate immunoproteasome subunits and acute chemokines within SCs to promote axonal and myelin debris clearance and macrophage recruitment [44].

To further corroborate these findings, we determined the release of IL-1β and VEGF at various time points of culture. For instance, IL-1β plays a key role in the differentiation mechanisms of SCs and is involved in the recruitment of immune cells, such as neutrophils and pro-inflammatory M1 macrophages [45,46]. We observed significantly elevated levels of IL-1β in the unconjugated bioscaffolds on days 7 and 9. These results suggest that tissue repair mechanisms are activated at an acute inflammatory timepoint, as high levels of IL-1β can inhibit regeneration and cause systemic inflammation [39,47]. Previous studies have established that MIP-1α can impede the release of IL-1β during highly inflammatory reactions, particularly in immune cells like macrophages, as secretion of this interleukin is highly dependent on MIP-1α mechanisms [47,48]. Since SCs are differentiating into a macrophage-like phenotype when activated by this model, we theorize that IL-1β mechanisms may similarly be “turned off” by the cells within this modeling system due to high levels of MIP-1α exposure and acquired immune cell-like behavior. Furthermore, there has been discourse about the existence of an IL-1β feedback loop following high inflammation, suggesting that SCs are not adopting a hyperinflammatory or cytotoxic phenotype in response to MIP-1α-conjugated bioscaffolds [48]. The reduction in IL-1β levels paired with an increase in VEGF highlights the selective amplification of pro-angiogenic and pro-repair signaling necessary for tissue bridge formation. These neurovascular interactions are well documented. Mechanistic in vivo studies have established that polarized blood vessels serve as essential migratory tracks for SCs during regeneration [49], while biomimetic in vitro platforms have utilized SCs to actively pattern and stabilize microvascular networks [50]. While these detected factors are associated with immunomodulation and angiogenesis, subsequent in vitro and in vivo studies are needed to validate their actual functional impact on tissue repair.

Taken together, these results indicate that the MIP-1α-conjugated bioscaffolds drive an acute inflammatory SC response geared toward pro-regenerative tissue remodeling. Because this study concluded at day 9, these data capture a critical early window in SC biology characterized by an acute phase response. Throughout this culture period, SCs persistently release chemotactic factors intended to recruit neutrophils, macrophages, and T cells for myelin debris clearance, alongside paracrine signals meant to stimulate endothelial cells to initiate neurovascular remodeling. Crucially, the cells promote this microenvironment by suppressing anti-inflammatory signals (e.g., IL-13 and IL-10) to allow for thorough debris removal and also chronic inflammatory markers like IL-1β to prevent cytotoxicity. While the secretome profiles strongly imply a pro-repair environment, the actual ability to recruit these cells and the complex paracrine crosstalk between SCs and other cell types within the multicellular milieu will need to be rigorously examined in future studies. A full characterization of this distinct genetic shift will also require tracking classical molecular and transcriptional markers, such as upregulation of c-Jun and p75NTR, alongside long-term neurotrophic drivers such as GDNF and BDNF.

The neuro-immune microenvironment can be beneficial or detrimental to nerve repair depending on the timing of its activation and the intensity of its response. In the acute aftermath of an injury, cytokines and chemokines initiate vital reparative responses, activate SCs, regulate Wallerian degeneration, and support early nerve recovery. A prime example of this temporal precision is MIP-1α, which increases within 24 h post-injury and peaks precisely on day 5. This acute peak serves as an essential chemotactic driver for SC cords to migrate efficiently across the injury site to form a cellular bridge, an effect that is independent of bridge vascularization or local macrophage density [15,35]. Furthermore, specific chemokines such as CXCL5, CXCL12, and CCL2 can act directly on neurons and axons during this early phase to accelerate axonal regeneration.

Studies have reported poor regenerative outcomes due to both a delayed initial immune response and a persistent, hyper-inflammatory state characterized by chronic macrophage retention and sustained inflammatory cytokine expression [51]. Directly tied to these temporal shifts are the quantitative thresholds of localized signaling molecules. For instance, optimal concentrations of pro-inflammatory cytokines are required to stimulate axonal growth and to achieve recovery of functional innervation after nerve injury. Completely neutralizing these early pro-inflammatory cytokines impairs peripheral nerve regeneration [45]. Conversely, the chronic overproduction of cytokines shifts the injured microenvironment into a pathological state, directly implicating these factors in the induction of neuropathic pain and subsequent regenerative failure [52]. Overall, these studies suggest that while an attenuated immune response impedes the essential tissue-clearing process that must precede new growth, an unchecked, high-intensity hyper-inflammatory state stalls functional tissue bridging. In this research, SCs showed elevated secretion of chemotactic, inflammatory, and angiogenic signaling, capturing the acute phase of the regenerative process. Evaluating longer culture time points and downstream upregulation of neurotrophic factors in future studies will determine the regenerative potential of SCs exposed to MIP-1α-conjugated bioscaffold.

Our results also show that SC-conditioned media from the conjugated bioscaffolds did not inhibit or impair neurite outgrowth compared to the unconjugated control. Studies have shown that high concentrations of inflammatory cytokines can often exert neurotoxic or inhibitory effects on axonal growth [53]. The observation that neurite extension remained unaffected indicates that the secretory profile induced by MIP-1α-conjugated bioscaffold does not compromise neuronal activity. This establishes the platform as a stable, biocompatible microenvironment suitable for further evaluation in multi-lineage tissue repair applications.

In this study, the primary SC response to the MIP-1α-conjugated scaffolds was similar to that of the SC line. Both models demonstrated robust spheroid retention and an upregulation of pro-inflammatory factors. However, due to the limited cell yields and the batch-to-batch heterogeneity associated with primary cell isolations, we elected to proceed with a well-established SC line. This approach ensured experimental reproducibility and statistical power across all assays.

Future studies will investigate longer time points to assess long-term bioscaffold degradation and its impact on SC activity. By integrating endothelial cells and/or motor neurons into the culture system alongside SCs [49,50], this platform can help unravel the complex cellular and molecular crosstalk that drives peripheral nerve repair. Future iterations of this model can also incorporate conductive nanomaterials or biopolymers that can synergize with electrical stimulation-based therapeutics for nerve repair [54]. Ultimately, this model serves as a highly adaptable foundational model for next-generation biomimetic environments.

## 3. Conclusions

In summary, we have developed a biomimetic 3D platform that successfully integrates native ECM composition, directional topographies, and localized chemokine signaling. Because native ECM molecules like laminin and collagen are preserved within our lyophilized hydrogel, the conjugated MIP-1α works synergistically with these native cell-adhesive proteins to stabilize dense spheroidal cellular structures and enhance their proliferative and migratory capacity. It was also shown that the acute inflammatory environment induced by MIP-1α conjugation did not hinder neurite extension, further corroborating the adoption of a reparative role in SCs. Ultimately, this integrated system serves as a valuable in vitro tool for studying SC-mediated peripheral nerve repair in response to various neurotrophic factors and cytokines.

## 4. Materials and Methods

### 4.1. Bioscaffold Synthesis

Autoclaved deionized (DI) water was heated in a water bath to 75 °C to make a 3 wt% porcine skin gelatin (Sigma Aldrich, St. Louis, MO, USA) solution. After the solution was completely dissolved, 21 mg of EDC (Sigma Aldrich, St. Louis, MO, USA) and 9.66 mg of NHS (Sigma Aldrich, St. Louis, MO, USA) were added. Rat tail collagen I (11.61 mg/mL; BD Corning, Corning, NY, USA) was diluted to a concentration of 3 mg/mL in 1× phosphate-buffered saline (PBS; Cytiva, South Logan, UT, USA). In a 24-well plate, 1.47 mL of the crosslinked gelatin solution was combined with 630 μL of the collagen solution and 17.5 μL of LM-111 solution (6 mg/mL; R&D Systems, Minneapolis, MN, USA). The final concentrations of the components were 21 mg/mL of gelatin, 0.9 mg/mL of collagen, 50 μg/mL of LM-111, 14.6 mM of EDC, and 5.6 mM of NHS. The solution was polymerized in a refrigerator (4 °C) for 1 h to form a hydrogel and then froze overnight at −8 °C. The well plate was then moved to −80 °C for a minimum of 72 h before lyophilization for 12–24 h. The lyophilized scaffolds were frozen at −20 °C until needed.

### 4.2. GelGroover Design

**Figure 9:**
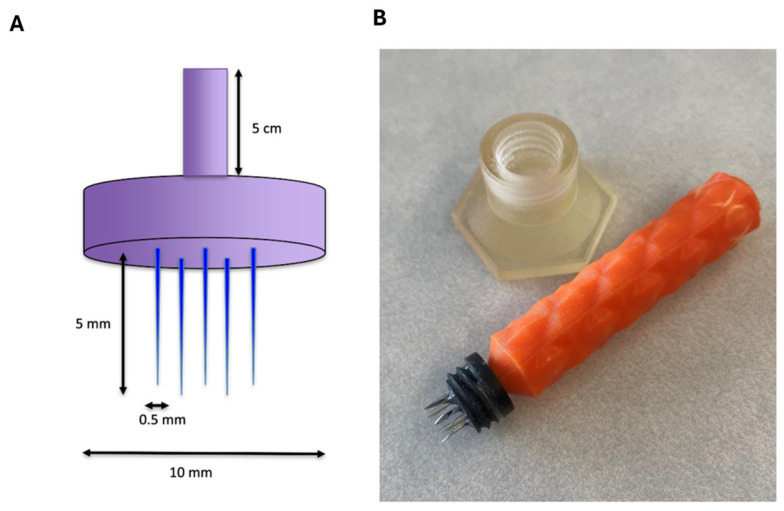
(**A**) Schematic of GelGroover device design and (**B**) the GelGroover device.

To create longitudinal channels through the bioscaffold, a custom device was designed. As channel formation is a highly mechanical process, it was necessary to create a 3D printed device that would ensure accuracy and reproducibility of channels for experimentation purposes. The GelGroover device was made in collaboration with SLU Center for Additive Manufacturing (Saint Louis University, St. Louis, MO, USA), following specifications in diameter, probe height, and probe width (Figure 9). The device was designed to be easily sterilized for stamping channels throughout the bioscaffold height.

### 4.3. Channel Formation and Scaffold Sterilization

Bioscaffolds were thawed at room temperature (RT) for 30 min, then sterilized with 70% ethanol and biopsy punched into a cylindrical geometry with a diameter of 10 mm. Bioscaffolds were moved to a 48-well plate for channel formation. The custom-made GelGroover device was sterilized with 70% ethanol and rinsed with 1× PBS. The GelGroover device was used to stamp 5 evenly spaced, longitudinal channels through ~70% of the height of the bioscaffold to form the BioGrooved scaffolds. Afterward, the BioGrooved scaffolds were rinsed twice with 1× PBS for 5 min and were allowed to dry at RT in the cell culture hood. Freshly lyophilized BioGrooved scaffolds were characterized using SEM (Saint Louis University, St. Louis, MO, USA). Select bioscaffolds were cut transversely to observe channel depth. The BioGrooved scaffolds were sputter coated for 3 min (SCD 005, Bal-Tec, Canonsburg, PA, USA). SEM imaging was performed at 2 kV to avoid sample burning. Channel diameter and depth were approximated using the accompanying SEM software and used to verify successful channel formation prior to cellular seeding.

### 4.4. Formation of Functionalized Chemotactic Region

To form the crosslinking solution, 19.17 mg of EDC and 5.75 mg of NHS were added to 20 mL of sterile 1× PBS. Aliquots of MIP-1α (0.01mg/mL, PeproTech, Cranbury, NJ, USA) were thawed on ice. In two microcentrifuge tubes, 1 mL of EDC/NHS crosslinking solution was added. In one of the tubes, 5 μL of MIP-1α was added to form a 50 ng/mL MIP-1α/EDC/NHS conjugate crosslinking solution. In a new 48-well plate, 100–200 μL of the select crosslinking solutions were added (*n* = 5 wells MIP-1α/EDC/NHS solution, *n* = 5 wells EDC/NHS solution). Thoroughly dried, sterilized BioGroove scaffolds were placed channel-side down in the wells containing crosslinking solutions and were allowed to soak in the incubator for 30 min to form the chemotactic region through absorption. The bioscaffolds were then flipped to be channel-side up and rinsed with 1× PBS for 5 min to remove unreacted EDC and NHS.

### 4.5. Cell Culture and Spheroid Formation

For primary cell isolation, the sciatic nerves of 10–12-week-old Sprague Dawley rats were harvested following sacrifice. The sciatic nerves were thoroughly cleaned of all adipose and connective tissue debris in a large Petri dish with 1× PBS. A razor blade was used to carefully bisect the nerves transversely, then the nerve halves were fragmented further into 3–5 mm long sections. A 6-well plate was coated with a 1% Matrigel (Matrigel Matrix, 21.56 mg/mL; 354248, Corning, Corning, NY, USA) solution in 1× PBS overnight. The nerve fragments were transferred into the prepared 6-well plate with warm proliferation media containing Dulbecco’s Modified Eagle Medium with Ham’s F12 (DMEM-F12; Cytiva, South Logan, UT, USA), 10% Fetal Bovine serum (FBS; Sigma Aldrich, St. Louis, MO, USA), and 1% Penicillin–Streptomycin (P/S; 100×, Gibco, Waltham, MA, USA). The plate was monitored and cultured at 37 °C (5% CO_2_) until cells attached to the coating. Once the cells were ~60% confluent, the nerve fragments were removed. Then, the cells were passaged and transferred to a 6-well plate prepared with a LM-111 (50 µg/mL in 1× PBS overnight at 4 °C) and poly-D-lysine hydrobromide (PDL; 100 µg/mL for 3 h at RT; A-003-E, Sigma Aldrich, St. Louis, MO, USA) coating for continued culture. The wells were monitored and passaged until a predominately (>80%) SC population was reached, which was verified via s100β antibody (ab52642; Abcam, Cambridge, UK) and DAPI staining (1:5000; Invitrogen, Waltham, MA, USA).

Schwann cells (SCs; RT4-D6P2T murine Schwannoma Cells, ATCC, Manassas, VA, USA) were cultured at 37 °C (5% CO_2_) in a T75 flask with warm proliferation media until 80% confluent. Prior to spheroid formation, a small amount of SCs were cultured on a 24-well plate, then stained with s100β antibody and DAPI to verify cell identity and ensure homogeneity.

An Aggrewell-400^TM^ well plate (STEMCELL Technologies, Vancouver, BC, Canada) was prepared according to the manufacturer’s protocol using anti-adherence solution (STEMCELL Technologies, Vancouver, BC, Canada) and rinsed twice with warm proliferation media. Then, 1 mL of warm proliferation media was added to each prepared well and placed in the incubator until usage. SCs were trypsinized (Trypsin; Gibco, Waltham, MA, USA) and resuspended to a density of 720,000 cells/100 µL. Immediately after resuspension, the prepared Aggrewell-400^TM^ plate was removed from the incubator, and 100 µL of cell suspension was added to each well to achieve a density of 600 cells/spheroid, based on results from a previous study [55]. Another 0.9 mL of proliferation media was added to each well to achieve a total volume of 2 mL, and the plate was centrifuged at 100× *g* for 5 min to form micro-aggregates. The well plate was then transferred to the incubator on a rotary rocker and allowed to culture for three days to form compact cellular spheroids. Spheroid formation was checked using brightfield microscopy to verify successful formation prior to cellular seeding.

### 4.6. Scaffold Culture

SC spheroids were removed from the microwells via vigorous pipetting and media washing per manufacturer’s protocol. The spheroids were collected in a 50 mL conical tube following each media wash and centrifuged at 200× *g* for 5 min. Then, SC spheroids were gently resuspended in proliferation media to a density of 1200 spheroids/50 µL of suspension. Each channel of the chemotactic and control BioGroove scaffolds were seeded directly with 10 µL of spheroid cell suspension to achieve a density of 1200 spheroids/bioscaffold. The spheroids were allowed to attach to the BioGroove scaffolds in the incubator for 15–20 min, then an additional 500 µL of proliferation media was added to supply nutrients, and the plate was placed into the incubator for culture. After 24 h, supernatant was collected in labeled microcentrifuge tubes, then the seeded bioscaffolds were moved to new wells and flipped horizontally to further promote cell dispersion. New proliferation media was added to all wells (former and current) and the bioscaffolds continued to culture horizontally. Thereafter, supernatant was collected every other day during feedings from each well and placed in labeled microcentrifuge tubes for later analysis. The seeded BioGroove scaffolds were cultured for a total of nine days, then cryopreserved in histomolds using optimal cutting temperature compound (OCT; Scigen, Houston, TX, USA).

### 4.7. BioGroove Scaffold Histological Analysis Techniques

The preserved bioscaffolds were cryosectioned (Leica CM1850; Lecia Biosystems, St. Louis, MO, USA) on charged slides for both primary (*n* = 3–5 scaffolds/group) and cell-line (*n* = 5 scaffolds/group)-derived SCs. Cell-seeded bioscaffold cryosections were stained and cover slipped in a single step using Vectashield with DAPI (Vector Laboratories, Newark, CA, USA). The slides were imaged using fluorescent microscopy (Axiovert 200M; Zeiss, Oberkochen, Germany) at 5×, 10×, and 20× magnifications, ensuring no cellular overlap between images for analysis. DAPI Area fraction was determined for each 10× magnification image using the Area Fraction Analysis and a thresholding between 30–40 units on ImageJ (FIJI, GitHub, version 1.54k). Spheroid quantification and characterization were determined for each 5× magnification image using the Particle Analysis (2000-Infinity, pixel units) and a thresholding between 30–40 units on ImageJ. DAPI Area Fraction Percent (%) and Spheroid Count were plotted in GraphPad PRISM version 10.0.0 (GraphPad, Boston, MA, USA) for statistical analysis.

For cellular distribution analysis, the 5× magnification channel images were stitched together to form complete representations of cell-line channel cross-sections (*n* = 4 representations/group). These images were transformed into 16-bit representations and processed using the Oval Profile Plugin (Bill O’Connell, 2015, attbi.com) in ImageJ by encapsulating the spheroid channels with the oval tool. This allows the alignment of spheroids within the channels to be represented by relative intensity peaks (Gray Value) through Fast Fourier Transform analysis (FFT). The relative intensity FFT data was plotted using Excel for comparison. Additionally, the FFT data was converted to full width at half maximum values (FWHM) of the central maximum point in Excel utilizing an established programming guide (“How to Calculate FWHM in Excel” by Spencer Lanoue, Bricks, 2025) and removing noise (*n* = 3–4 peaks/group).

A select group of SC spheroids cultured for four days were stained with Live/Dead^TM^ staining (Life Technologies Corporation, Eugene, OR, USA) and imaged at 5× magnification using fluorescent microscopy (*n* = 5 wells). Spheroids were quantified using ImageJ (Particle Analysis, 2000-Infinity) and the number of dead cells was also quantified (Particle Analysis, 0-Infinity). The total number of cells per field of view (PFV) was determined by multiplying spheroid count by the number of cells per spheroid (600 cells/spheroid). The percentage of dead cells and living cells was manually determined using Excel for each image and averaged to determine spheroid viability. Spheroid diameter was quantified from the Live/Dead stain images using scaling on ImageJ. Spheroid Viability and Spheroid Diameter were plotted in GraphPad PRISM version 10.0 for statistical analysis.

### 4.8. BioGroove Scaffold Biochemical Analysis Techniques

Cell culture supernatants collected on days 3, 5, 7, and 9 of culture were subjected to interleukin-1β (IL-1β) ELISA analysis (Kit 900-K91; PeproTech, Cranbury, NJ, USA) and VEGF ELISA analysis (Kit 900-K436K; PeproTech, Cranbury, NJ, USA) to determine expression throughout culture (*n* = 5 scaffolds/group). ELISA data was measured using a spectrophotometer (SpectraMax i3, Molecular Devices, San Jose, CA, USA) and its corresponding software (SoftMax Pro 6.3). Data collected from the software was processed through a customized MATLAB R2025b program to determine the desired standard fit, then was plotted in GraphPad PRISM version 10.0.0 for statistical analysis and visualization. A cytokine array on the primary SC spheroids (Proteome Profiler ^TM^ Array, ARY030, R&D Systems, Minneapolis, MN, USA) was performed on day 7 supernatants pooled from all the wells for each group (final volume: ~0.3 mL), as this represents the peak early inflammatory time point for cellular secretion. A cytokine array on the cell-line-derived SC spheroids (Proteome Profiler ^TM^ Array, ARY008, R&D Systems, Minneapolis, MN, USA) was performed on day 1 and day 7 supernatants pooled from all the wells for each group (*n* = 5 wells/group/day). Approximately 80 µL was pooled from each sample to obtain a final pooled volume of 0.4 mL for each time point and group, which was then added to the cytokine array. The intensity of positive expression demonstrated by the antibody dots on the membranes was quantified using the gel analysis feature on ImageJ. The dot intensities were plotted into peaks, and the area under each curve was measured to obtain relative values. A representative Log_2_ Fold Change was calculated from the intensity values using Excel [56], which was plotted in GraphPad PRISM version 10.0.0 for further analysis.

### 4.9. NSC-34 Extension Assay

Neural stem cells (Mouse Motor Neuron-Like Hybrid Cell Line (NSC-34; RRID:CVCL_D356; CLU140, Cedarlane Cellutions Biosystems Inc., Burlington, ON, Canada) were differentiated in response to collected experiment secretome. NSC-34 cells were cultured in a T75 flask with proliferation media (DMEM-F12 with 10% FBS and 1% P/S) to 60% confluency. Cells were trypsinized and resuspended to a density of 1 × 10^6^ cells/mL for plating. In a 96-well plate, 1 µL of cell suspension (~1000 cells) was added to 100 µL of proliferation media (*n* = 5 wells/group) for each of the three experimental groups. NSC-34s were allowed to culture in proliferation media for 24 h, then were differentiated using differentiation media based on experimental groupings. The positive control group was differentiated in regular differentiation media containing DMEM-F12, 1% FBS, 1% P/S, 1% Minimum Essential Medium Non-essential Amino Acids (MEM NEAA; 100×; Gibco, Brooklyn, NY, USA), and 1 µM retinoic acid (RA; 50 mM in dimethyl sulfoxide). The non-MIP-1α and MIP-1α groups were cultured with a mix of regular differentiation media and collected D9 supernatant (no conjugation vs. conjugation) in a 1:1 ratio. The cells were differentiated for 72 h with 100 µL of new differentiation media exchanged each day of culture. Brightfield microscopy was used to image the wells each day of differentiation for quantification in ImageJ of neurite extension length. Extension data was plotted in GraphPad PRISM version 10.0.0 for statistical analysis.

### 4.10. Data Analysis and Processing

All statistical analysis and graphing were performed in GraphPad Prism version 10.0.0. For ELISA data, a two-way ANOVA was performed to assess statistical significance. To determine the statistical significance of DAPI area fraction, spheroid count, and FWHM datasets, a two-tailed Student’s *t*-test analysis was performed. To analyze spheroid viability data, an unpaired *t*-test analysis was performed. Outlier identification (ROUT) was performed on PRISM for all datasets.

## Data Availability

All data are included in the manuscript. Additional information is available upon request.

## Abbreviations

The following abbreviations are used in this manuscript:

PNS	Peripheral Nervous System
PNI	Peripheral Nerve Injury
LPNI	Laceration Peripheral Nerve Injury
SC	Schwann Cells
MIP-1α	Macrophage Inflammatory Protein-1α
BNC	Biomaterial Nerve Conduit
ECM	Extracellular Matrix
EDC	1-ethyl-3-(3-dimethylaminopropyl) carbodiimide
NHS	N-hydroxysuccinimide
SEM	Scanning Electron Microscopy
PFV	Per Field of View
FFT	Fast Fourier Transform
VEGF	Vascular Endothelial Growth Factor
IL-1β	Interleukin-1β
RANTES	Regulated on Activation Normal T-Cell Expressed and Secreted
IL-6	Interleukin-6
GM-CSF	Granulocyte–Macrophage Colony-Stimulating Factor
CINC-1	Cytokine-induced Neutrophil Chemoattract 1
IL-13	Interleukin-13
IL-17	Interleukin-17
RT	Room Temperature
NSC-34	Nerve Stem Cells-34
